# Ectopic expression of tea *MYB* genes alter spatial flavonoid accumulation in alfalfa (*Medicago sativa*)

**DOI:** 10.1371/journal.pone.0218336

**Published:** 2019-07-02

**Authors:** Guangshun Zheng, Cunying Fan, Shaokang Di, Xuemin Wang, Liping Gao, Nikolay Dzyubenko, Vladimir Chapurin, Yongzhen Pang

**Affiliations:** 1 Institute of Animal Science, Chinese Academy of Agricultural Sciences, Beijing, China; 2 Key Laboratory of Plant Resources and Beijing Botanical Garden, Institute of Botany, the Chinese Academy of Sciences, Beijing, China; 3 School of Life Science, Anhui Agriculture University, Hefei, China; 4 Federal Research Center of Russian Vavilov Institute of Plant Genetic Resources, St. Petersburg, Russia; Texas Tech University, UNITED STATES

## Abstract

Flavonoids are one of the largest secondary metabolite groups, which are widely present in plants. Flavonoids include anthocyanins, proanthocyanidins, flavonols and isoflavones. In particular, proanthocyanidins possess beneficial effects for ruminant animals in preventing lethal pasture bloat. As a major legume forage, alfalfa (*Medicago sativa*) contains little proanthocyanidins in foliage to combat bloat. In an attempt to improve proanthocyanidin content in alfalfa foliage, we over-expressed two MYB transcription factors (*CsMYB5-1* and *CsMYB5-2*) from tea plant that is rich in proanthocyanidins. We showed that, via targeted metabolite and transcript analyses, the transgenic alfalfa plants accumulated higher levels of flavonoids in stems/leaves than the control, in particular anthocyanins and proanthocyanidins. Over-expression of *CsMYB5-1* and *CsMYB5-2* induced the expression levels of genes involved in flavonoid pathway, especially anthocyanin/proanthocyanidin-specific pathway genes *DFR*, *ANS* and *ANR* in stems/leaves. Both anthocyanin/proanthocyanidin content and the expression levels of several genes were conversely decreased in flowers of the transgenic lines than in control. Our results indicated that *CsMYB5-1* and *CsMYB5-2* differently regulate anthocyanins/proanthocyanidins in stems/leaves and flowers. Our study provides a guide for increasing anthocyanin/proanthocyanidin accumulation in foliage of legume forage corps by genetic engineering. These results also suggest that it is feasible to cultivate new varieties for forage production to potentially solve pasture bloat, by introducing transcription factors from typical plants with high proanthocyanidin level.

## Introduction

Flavonoids, including anthocyanins, proanthocyanidins, flavonols and isoflavones, are important secondary metabolites that are widely present in plants. Flavonoids play vital roles in defending against pathogens and herbivorous animals, protecting against UV damage, attracting pollination, and changing the color of flowers [1–6]. Among them, proanthocyanidins have been increasingly found to be economically important in phytochemical compound and food for human health and in feed for ruminant animals [7–9]. Proanthocyanidins can effectively eliminate pasture bloat, improve the conversion efficiency of forage proteins into animal proteins, reduce greenhouse gases [10]. Therefore, it is of great economic value to improve proanthocyanidin content in forage crops, like alfalfa that hardly accumulates proanthocyanidins in foliage.

Anthocyanins and proanthocyanidins share the upstream biosynthetic pathway and they are regulated by a number of transcription factors. Among them, a MBW ternary complex, including MYB, bHLH and WD40 regulatory proteins, regulates tissue-specific accumulation of anthocyanins/proanthocyanidins [11]. In particular, the MYB transcription factors provide specificity to this MBW complex [12]. The two well-documented MYB transcription factor genes in *Arabidopsis thaliana* are *Production of Anthocyanin Pigmentation* (*PAP1*) and *TT2* (*AtMYB123*) in the regulation of anthocyanidins and proanthocyanidins, respectively. *PAP1* specifically regulates biosynthetic pathway genes *DFR* and *ANS* for anthocyanin accumulation in leaves of *A*. *thaliana* [13–14]. TT2 in the MBW ternary complex functions as a master regulator of proanthocyanidin biosynthesis in seeds, by activating proanthocyanidin-specific pathway gene *ANR* [11,15].

TT2 homologs involved in proanthocyanidin biosynthesis were also identified in other plant species, such as *Lotus japonicas* (LjTT2a, LjTT2b and LjTT2c) [16], *Trifolium arvense* (TaMYB14) [17], *Diospyros kaki* (*DKMYB4*) [18], and grape (*VvMYBPA1*, *VvMYBPA2* and *VvMYB5*) [19–21]. In the model legume plant *Medicago truncatula*, a close relative of alfalfa, the MYB transcription factor *MtPAR* was found to be involved in proanthocyanidin regulation in seeds [22]. MYB5 and MYB14 from *M*. *truncatula* were synergistic in regulating the expression levels of *ANR* (anthocyanidin reductase), *LAR* (leucoanthocyanidin reductase) and proanthocyanidin biosynthesis pathway genes in the presence of *MtTT8* and *MtWD40-1* [23]. These results suggested that MYB transcription factors play important roles in proanthocyanidin biosynthesis.

As a major legume forage crop worldwide, alfalfa contains little proanthocyanidins in foliage. Therefore, over-expression of *MYB* transcription factor genes that regulate proanthocyanidin accumulation is an ideal strategy for genetic improvement of alfalfa varieties with improved proanthocyanidin content [2]. In a previous study, the maize transcription factor genes *B-Peru* and *C1* were transferred into alfalfa, but proanthocyanidin level was not increased in the leaf tissue [24]. Other positive regulatory genes were transformed into alfalfa, including *MtLAP1* [25], *MtWD40-1* [26], *MtPAR* [22], but none of them succeeded in producing significant amount of proanthocyanidins to combat bloat. Proanthocyanidin content was much lower than expected in those transgenic alfalfa plants, mainly because endogenous gene *ANR* was not significantly up-regulated [22, 25, 26]. Although *MYB* transcription factor genes *TT2* and *MtPAR* can activate proanthocyanidin accumulation in hair roots of *M*. *truncatula* [22, 26], alfalfa plants over-expressing *TT2* did not promote the production of proanthocyanidins in alfalfa foliage [25]. Therefore, it is necessary to explore additional candidate genes for proanthocyanidin bioengineering.

Tea plant is rich in flavonoid compounds, in particular proanthocyanidins and its precursor flavanols [27, 28]. In a previous study, a number of *MYB* transcription factor genes were identified to be potentially involved in proanthocyanidin regulation in tea plant. Among them, *CsMYB5-1* and *CsMYB5-2* showed high similarity with *TT2*, *C1*, *DKMYB4* and *VvMYB5* [29], and their expression levels correlated with the accumulation level of proanthocyanidins [28]. *CsMYB5-1* and *CsMYB5-2* are thus ideal candidate genes for proanthocyanidin improvement in alfalfa by genetic engineering. In the present study, *CsMYB5-1* and *CsMYB5-2* were respectively over-expressed in alfalfa by *Agrobacterium*-mediated transformation, driven by the cauliflower mosaic virus (CaMV) 35S. The over-expression of *CsMYB5-1* and *CsMYB5-2* increased total flavonoid, anthocyanin and soluble proanthocyanidin contents in stems/leaves, with the up-regulation of a number of pathway genes. Interestingly, over-expression of *CsMYB5-1* and *CsMYB5-2* in alfalfa led to decreased total flavonoid, anthocyanin and soluble proanthocyanidin contents in flowers. Our results demonstrated that *CsMYB5-1* and *CsMYB5-2* differently regulate flavonoid accumulation in stems/leaves, and flowers, and they are potential candidate genes for modification of flavonoid compounds in alfalfa by genetic breeding.

## Materials and methods

### Vector construction and plant transformation

The open reading frame regions of both *CsMYB5-1* (accession number: KY827396) and *CsMYB5-2* (accession number: KY827400) were amplified with cDNAs from young leaves of tea plants (cultivar “Shuchazao”). The PCR products were then inserted in the plant expression vector PCB2004 driven by the cauliflower 35S promoter as previously reported [27]. These two vectors were further introduced into *Agrobacterium tumefaciens* strain GV3101 for the transformation of alfalfa plants, respectively.

Young leaves of alfalfa (*Medicago sativa* L. cultivar Zhongmu No. 1) were used as explants via a protocol as described by Cosson *et al*. with minor modifications [30]. Callus after induction was transferred onto the fresh SH9 medium with 150 mg/L kinetin for plant regeneration. The generated seedlings were transferred onto a fresh SH9 medium containing 500 mg/L indole-3-acetic acid (IAA) for rooting. The phosphinothricin (ppt) concentration used for plant selection and rooting were 5mg/L. Alfalfa plants were grown at 24°C/22°C with 16/8 h light/dark cycles and a humidity of 60%.

### Identification of the transgenic alfalfa plants

Genomic DNAs from young leaves of the ppt-resistant lines were extracted by using a cetyltrimethyl ammonium bromide protocol [31]. All transgenic alfalfa plants were confirmed by PCR with forward primes CsMYB5-1F/CsMYB5-2F, and reverse primers CsMYB5-1R/CsMYB5-2R (S1 Table) and the following procedure: denatured at 94°C for 8 min, followed by 35 cycles of 94°C for 30 sec, 58°C for 45 sec and 72°C for 1 min, then extended at 72°C for 10 min.

The expression levels of *CsMYB5-1* and *CsMYB5-2* genes in the transgenic alfalfa plants were detected by RT-PCR. Total RNAs were extracted from the stems/leaves (two-month-old alfalfa plants) of PCR-positive lines and the wild type by using the TRIzol Kit (TianGen, China) according to the manufactures’ instruction. First-strand cDNA was synthesized by using HiFiScript Quick gDNA Removal cDNA Kit (CW Biotech, China) according to the instruction. The cDNA samples were amplified with the same sets of forward and reverse primes for *CsMYB5-1* and *CsMYB5-2* (S1 Table), the procedure was as followed: denatured at 94°C for 8 min, followed by 30 cycles of 94°C for 30 sec, 58°C for 45 sec and 72°C for 1 min, then extended at 72°C for 10 min. *ACTIN2* gene was used as internal control with forward and reverse primes (S1 Table). Both the PCR and RT-PCR products were separated on 1% agarose gel with ethidium bromide and visualized by ultraviolet gel imaging instrument.

qPCRs were performed by using UltraSYBR Mixture (CWBIO, Beijing, China) on a 7900 Real Time PCR System machine (Applied Biosystems, CA, USA). The reaction system of 20 μL contained 1 μL diluted cDNA (1:5) as template with RNAs extracted from stems/leaves (two-month-old alfalfa plants) and flowers (petal). The procedure was as followed: denatured at 95°C for 30 sec, followed by 40 cycles of 95°C for 5 sec and 60°C for 34 sec, then 95°C for 15 sec, 60°C for 1 min and 95°C for 15 sec. The primers of flavonoid pathway genes and internal control gene were listed in S1 Table. The relative expression levels of all genes were determined by using the 2^-ΔΔCT^ method and compared with those of the control. In the qPCR assay, three independent biological replicates were performed with technical triplicates, and data are presented as mean ± SD, student’s *t*-test (n = 3, *P<0.05, **P<0.01).

## Determination of the relative contents of anthocyanins, proanthocyanidins and total flavonoids

Anthocyanins from stems/leaves (two-month-old plants), and flowers (petals) of 10 mg samples (same as for RNA extraction) were extracted with 500 μL methanol containing 0.1% HCl, followed by the addition of the same amount of water and chloroform to remove chlorophyll. The anthocyanin contents in these samples were measured spectrophotometrically at 530 nm with cyanidin 3-*O*-glucoside as standard. The anthocyanin content in foliage and flowers of the wild type alfalfa were determined to be 7.18 ng/g dry weight, and 24.6 μg/g dry weight, respectively. The anthocyanin content in the transgenic lines were calculated and compared with the wild type as value of 1 in biological triplicate, and data are presented as mean ± SD, student’s *t*-test (n = 3, *P<0.05, **P<0.01).

Soluble proanthocyanidins were extracted from 50 mg dry samples three times with 1 mL extraction buffer (70% acetone containing 0.5% acetic acid), three times with chloroform, and twice with hexanes. The aqueous phase was then freeze dried and re-suspended in extraction buffer. The soluble proanthocyanidin levels were measured by heating in butanol/HCl (95:5) at 95°C for 1 h and detected at wavelength of 550 nm with procyanidin as standard [32]. The proanthocyanidin content in foliage and flowers of the wild type alfalfa were determined to be 0.53 mg/g dry weight and 3.26 mg/g dry weight, respectively. And proanthocyanidin content in the wild type control was set as value of 1.0. Similarly, the insoluble proanthocyanidins in these residues were also measured by using the butanol/HCl method. The content of the insoluble proanthocyanidins in stems/leaves and flowers in the wild type alfalfa were 0.25 mg/g dry weight and 0.36 mg/g dry weight, respectively. The proanthocyanidin level in the wild type was set as value of 1.0. All the assays were performed with triplicates and the data are presented as mean ± SD, student’s *t*-test (n = 3, *P<0.05, **P<0.01).

Total flavonoids were extracted from stems/leaves (10 mg dry weight) and flowers (5 mg dry weight) with 1 mL and 500 μL 80% methanol, respective. And 400 μL water, 30 μL 5% NaNO_2_, 30 μL 10% AlCl_3_, 200 μL 1M NaOH and 240 μL water were added to 100 μL of these extracts consequently. The absorptions of the final mixtures were measured spectrophotometrically at the wavelength of 510 nm with quercetin as standard. The total flavonoid content in stems/leaves and flowers of the wild type were determined to be 18.83 mg/g dry weight and 244.04 mg/g dry weight, respectively. Total flavonoid content was calculated with quercetin as standard, and the total flavonoid content in wild type was set as value of 1.0. All the assays were performed with triplicates and the data are presented as mean ± SD, student’s *t*-test (n = 3, *P<0.05, **P<0.01).

## Results

### Generation of the transgenic alfalfa plants over-expressing the *CsMYB5-1*/*CsMYB5-2* gene

**Fig 1:**
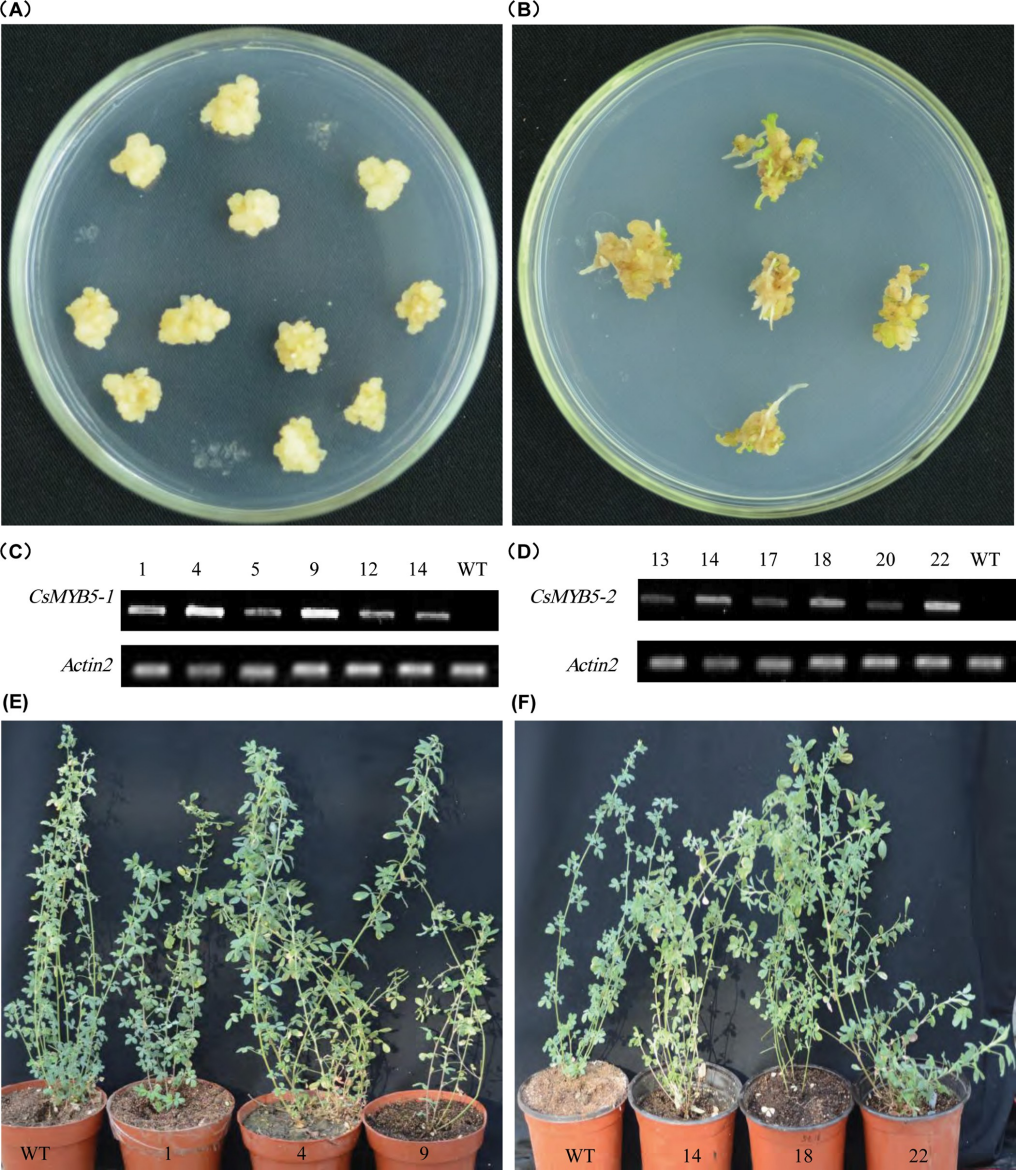
Generation of the transgenic alfalfa plants over-expressing *CsMYB5-1* and *CsMYB5-2* gene, respectively. (A) Generation of callus 30 days after infection; (B) Differentiation of the transgenic seedlings; (C-D) Expression levels of *CsMYB5-1* (C) and *CsMYB5-2* (D) in the transgenic alfalfa lines determined by RT-PCR analysis; (E-F) Generation of the transgenic alfalfa plants over-expressing *CsMYB5-1* (E) and *CsMYB5-2* (F).

To generate transgenic alfalfa plants, the PCB2004 vector containing coding sequence of the tea *CsMYB5-1* (930 bp) and *CsMYB5-2* (903 bp) gene were respectively introduced into alfalfa plants (*Medicago sativa* L. cultivar Zhongmu No. 1) via *Agrobacterium-*mediated transformation. Callus and regenerated shoots were screened on SH9 media containing phosphinothricin (Fig 1A and 1B). More than twenty independent phosphinothricin (ppt)-resistant transgenic lines were produced for each transgene, and the presence of the foreign genes were further confirmed by PCR analysis (S1 Fig).

To determine the expression levels of the *CsMYB5-1*/*CsMYB5-2* gene in the PCR-positive lines, RT-PCR was performed with gene-specific primes. It showed that *CsMYB5-1* and *CsMYB5-2* genes were expressed in the transgenic alfalfa plants at different levels, but not in the wild type plant (Fig 1C and 1D). These results indicated that *CsMYB5-1* and *CsMYB5-2* were successfully introduced and expressed in alfalfa plants. Subsequently, three independent transgenic alfalfa lines (Line Nos. 1, 4, 9 for *CsMYB5-1* and Line Nos. 14, 18, 22 for *CsMYB5-2*) that showed relatively high gene expression level were selected for further analyses (Fig 1C and 1D).

## Ectopic expression of the *CsMYB5-1*/*CsMYB5-2* gene promoted the accumulation of anthocyanins, total flavonoids and proanthocyanidins in alfalfa stems/leaves

**Fig 2:**
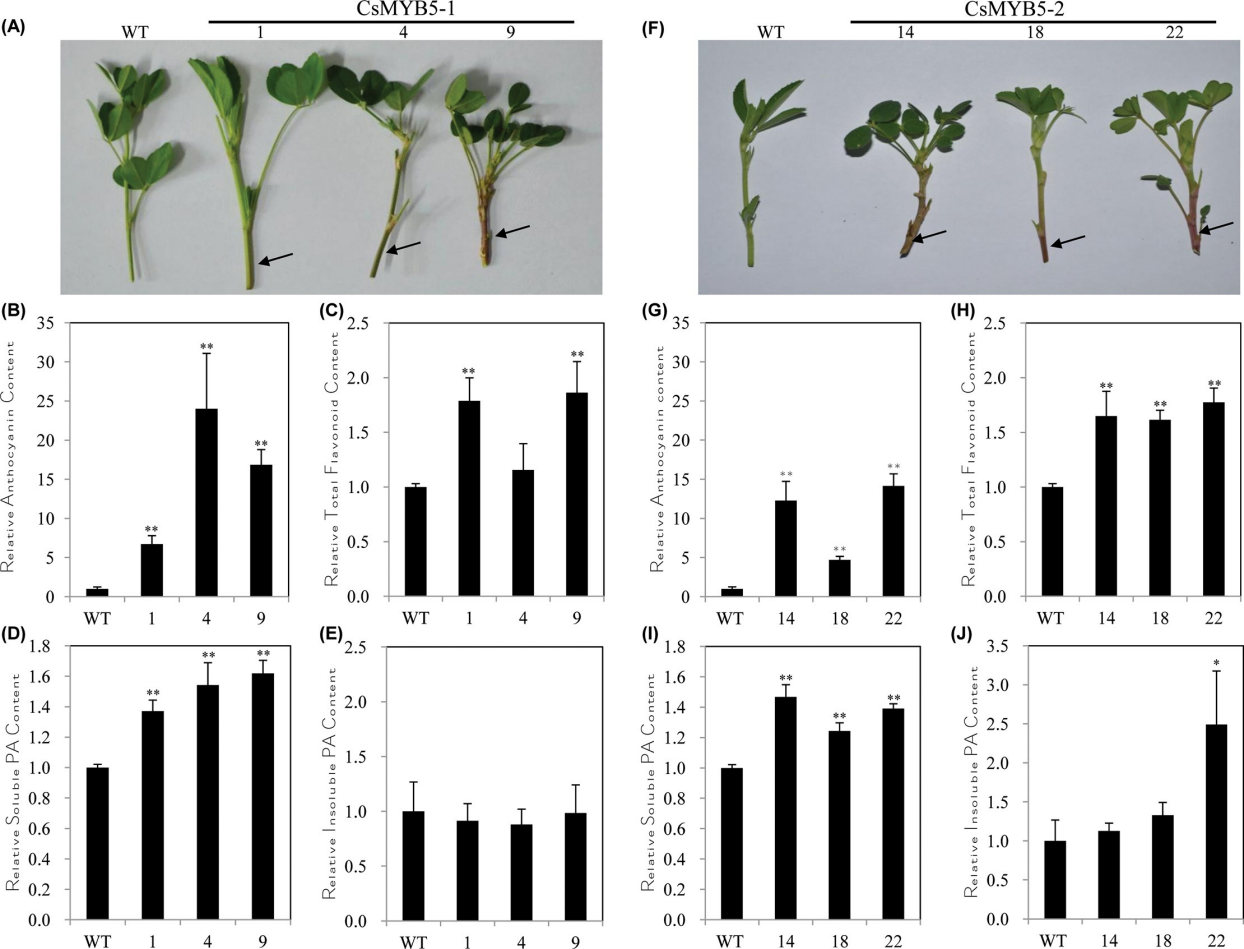
Morphology and flavonoid analyses in the foliage of the transgenic alfalfa plants over-expressing *CsMYB5-1*/*CsMYB5-2* gene, respectively. (A, F) Phenotype of the transgenic alfalfa plants (stems/leaves) over-expressing *CsMYB5-1* (A) or *CsMYB5-2* (F) in comparison with the wild type; (B, G) Relative total flavonoid content in the transgenic alfalfa plants over-expressing *CsMYB5-1* (B) or *CsMYB5-2* (G) in comparison with the wild type; (C, H) Relative anthocyanin content in the transgenic alfalfa plants over-expressing *CsMYB5-1* (C) or *CsMYB5-2* (H) in comparison with the wild type; (D, I) Relative soluble proanthocyanidin content in the transgenic alfalfa plants over-expressing *CsMYB5-1* (D) or *CsMYB5-2* (I) in comparison with the wild type; (E, J) Relative insoluble proanthocyanidin content in the transgenic alfalfa plants over-expressing *CsMYB5-1* (E) or *CsMYB5-2* (J) in comparison with the wild type. All the assays were performed with triplicates and the data are presented as mean ± SD, student’s *t*-test (n = 3, *P<0.05, **P<0.01).

There were no significant morphological changes between the transgenic and the wild type plants (Fig 1E and 1F). However, evident anthocyanin accumulation was found in the stems of the transgenic alfalfa plants, especially in the young stems (Fig 2A and 2F). These results demonstrated that *CsMYB5-1*/*CsMYB5-2* could promote the accumulation of anthocyanins in alfalfa, suggesting that they function regionally in the transgenic alfalfa plants. Total anthocyanin contents were increased by more than 6 fold and 4 fold in the *CsMYB5-1*/*CsMYB5-2* over-expressing lines (stems/leaves), respectively (Fig 2B and 2G, S2 Table). In particular, anthocyanin contents were increased by more than 10 fold in lines CsMYB5-1-4 and CsMYB5-2-22 (Fig 2B and 2G, S2 Table). Both phenotypic and quantitative analyses clearly demonstrated that *CsMYB5-1* and *CsMYB5-2* promote anthocyanin accumulation in stems/leaves of the transgenic alfalfa plants.

To further understand the effects of these two transcription factor genes on the accumulation of flavonoid compounds, the contents of total flavonoids and proanthocyanidins in stems/leaves of these transgenic lines were also quantified in comparison with the wild type. Total flavonoid contents were enhanced around 1.2–1.8 fold compared with the wild type (Fig 2C and 2H, S3 Table). Analysis on proanthocyanidin accumulation showed that only the soluble proanthocyanidin contents increased by 1.2–1.6 fold in both the *CsMYB5-1* and *CsMYB5-2* over-expressing alfalfa plants as compared to the wild type (Fig 2D and 2I, S4 Table), whereas no significant difference in insoluble proanthocyanidin content were observed between the transgenic lines and the wild type (Fig 2E and 2J, S4 Table), except for line CsMYB5-2-22.

Taken together, our results revealed that both *CsMYB5-1* and *CsMYB5-2* could enhance the accumulation of anthocyanins, total flavonoids and soluble proanthocyanidins, and they are potential candidate genes for the genetic engineering of flavonoids, in particular anthocyanins and proanthocyanidins, in alfalfa with increased quality traits.

## Effects of *CsMYB5-1*/*CsMYB5-2* on the expression of flavonoid pathway genes in alfalfa stems/leaves

**Fig 3:**
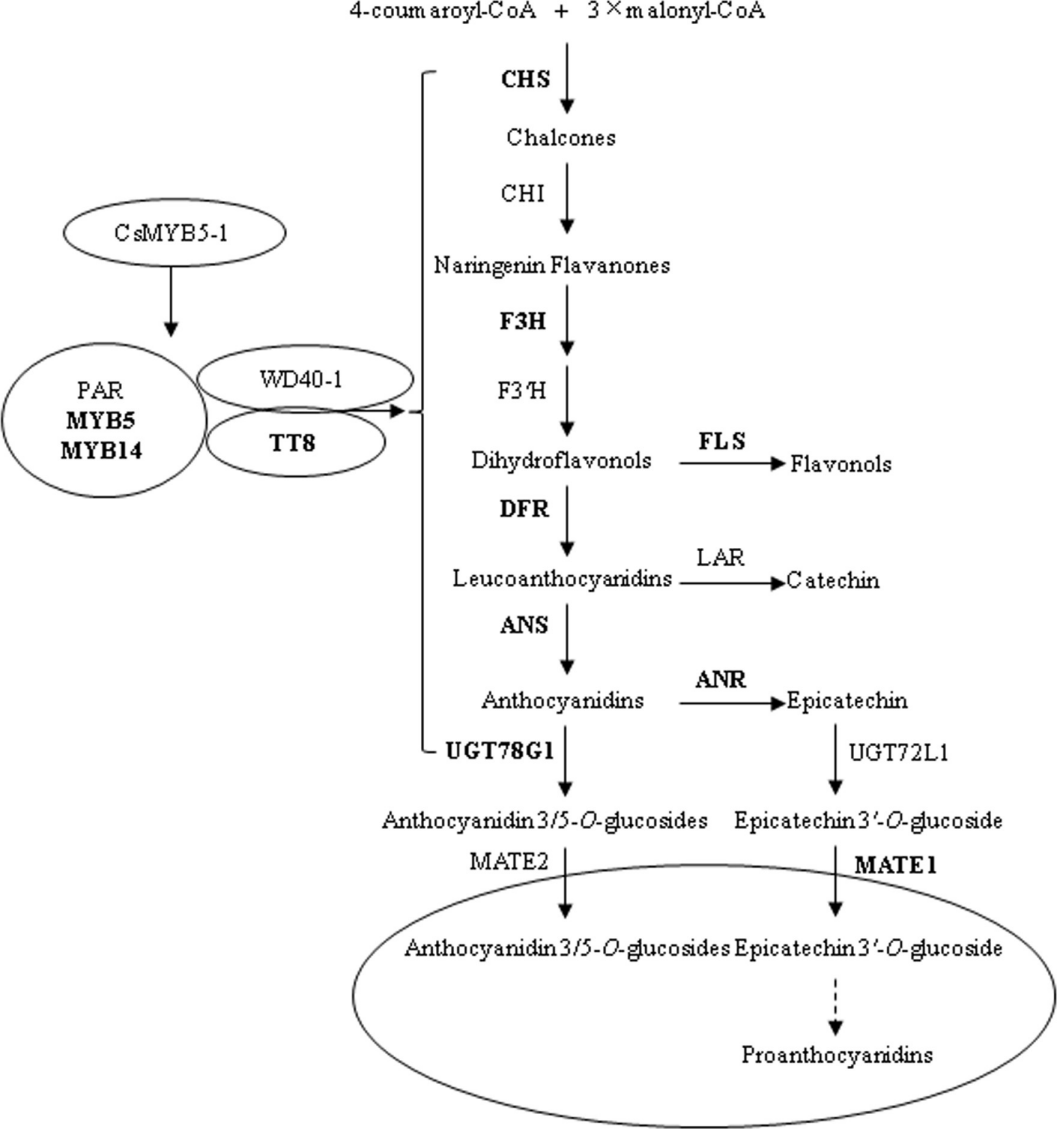
Proposed regulation mechanism of flavonoid biosynthetic pathway by *CsMYB5-1* in alfalfa. CHS, chalcone synthase; CHI, chalcone isomerase; F3H, flavanone 3-hydroxylase; FLS, flavonol synthase; DFR, dihydroflavonol 4-reductase; ANS, anthocyanidin synthase; ANR, anthocyanidin reductase; MATE1, multidrug and toxin extrusion transporter; UGT, UDP-glucose: flavonoid glycosyltransferase; TT8, transparent testa 8. Genes significantly regulated by CsMYB5-1 were bold.

**Fig 4:**
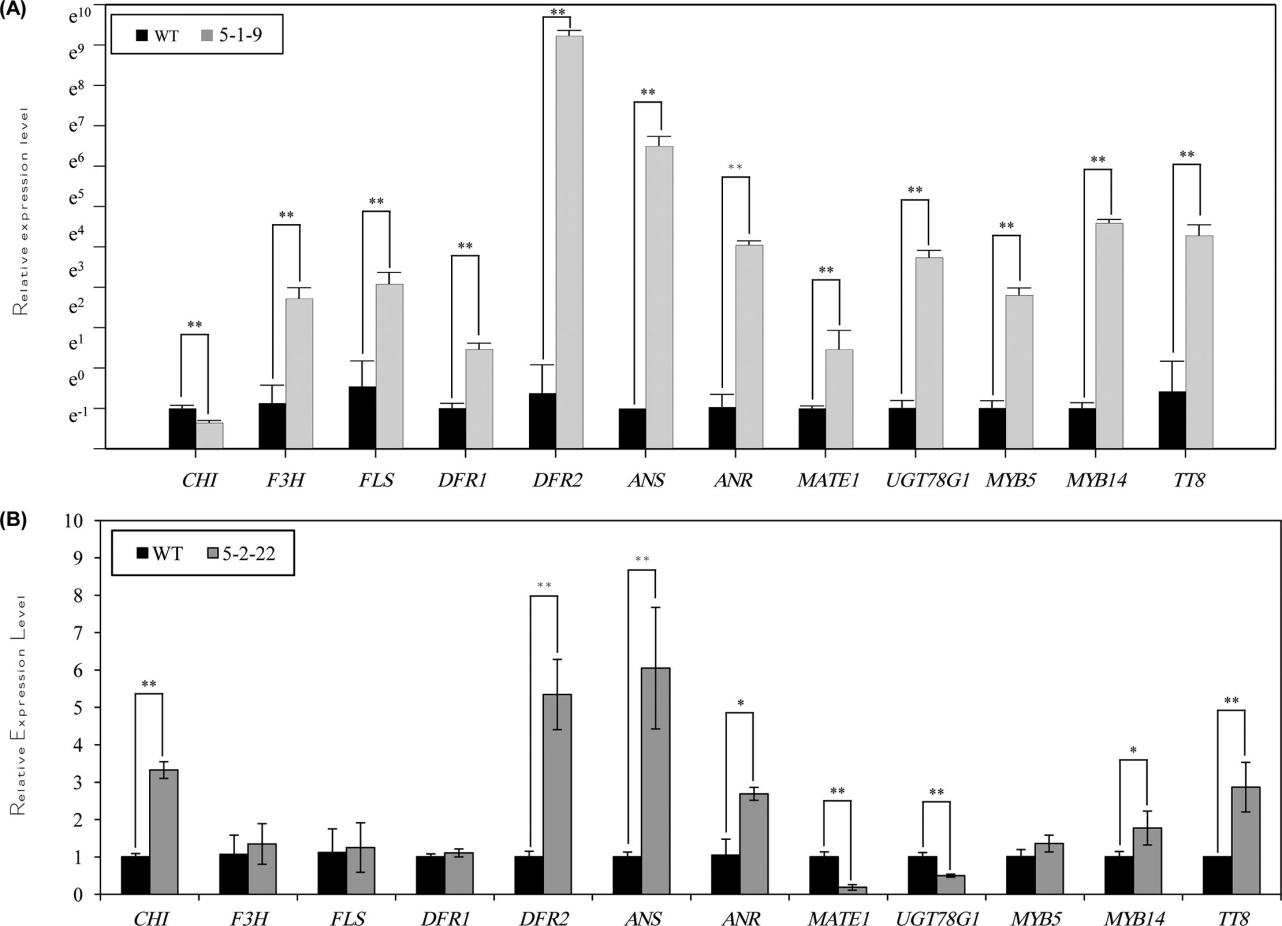
Relative expression level of flavonoid pathway genes in stems/leaves of the transgenic alfalfa lines over-expressing *CsMYB5-1*/*CsMYB5*, respectively. (A) Relative expression levels of flavonoid pathway genes in stems/leaves of the alfalfa lines over-expressing *CsMYB5-1* gene; (B) Relative expression levels of flavonoid pathway genes in stems/leaves of the alfalfa lines over-expressing *CsMYB5-2* gene. All the assays were performed with triplicates and the data are presented as mean ± SD, student’s *t*-test (n = 3, *P<0.05, **P<0.01).

To unravel the regulatory mechanisms of *CsMYB5-1*/*CsMYB5-2* gene in flavonoid pathway, the expression levels of several pathway genes were analyzed by qPCR in the two representative transgenic lines Nos. CsMYB5-1-9 and CsMYB5-2-22. The enzyme genes investigated were *CHI*, *F3H*, *FLS*, *DFR1*, *DFR2*, *ANS*, *ANR*, *MATE1* and *UGT78G1*, and transcription factor genes *MYB5*, *MYB14* and *TT8* (Fig 3). Among them, *CHI* and *F3H* respectively encode chalcone isomerase and flavanone 3-hydroxylase for the formation of naringenin chalcone and dihydroflavonols, *DFR*, *ANS* and *ANR* respectively encode dihydroflavonol 4-reductase, anthocyanidin synthase and anthocyanidin reductase for the formation of epicatechin via leucoanthocyanidin and anthocyanidin (Fig 3). *MATE1* encode a transporter protein that is specific for epicatechin 3'-*O*-glucoside, and *UGT78G1* encode a UDP-glucosyltransferase that is able to glycosylate anthocyanidin substrate. In the *CsMYB5-1* over-expressing line 5-1-9, the expression levels of all these tested genes (except *CHI*) increased at various levels compared with the wild type (Fig 4A, S5 Table), which were consistent with the elevated contents of anthocyanins, total flavonoids and proanthocyanidins. In particular, the expression levels of two enzyme genes *DFR2* and *ANS* were increased remarkably by more than 600 fold, indicating they are the major contributors for the increased anthocyanin content in stems/leaves. In addition, the expression levels of *MYB5*, *MYB14* and *TT8* were also highly up-regulated at various levels, indicating *CsMYB5-1* also up-regulates their expression levels for flavonoid accumulation (Fig 4B, S5 Table).

In the transgenic line *CsMYB5-2-22*, the expression levels of *CHI*, *DFR2*, *ANS*, *ANR*, *MYB14* and *TT8* were increased in comparison with the wild type (Fig 4B, S5 Table). In particular, the expression levels of *DFR2* and *ANS* were more than 4–5 fold higher than in the wild type (Fig 4B, S5 Table), which was consistent with the increase of anthocyanins, total flavonoids and proanthocyanidins. However, expression levels of these genes were much lower than those in the *CsMYB5-1-9* transgenic line, suggesting these endogenous genes were differentially regulated by *CsMYB5-1* and *CsMYB5-2*. These results demonstrated that *CsMYB5-1* and *CsMYB5-2* regulated the expression of flavonoid pathway genes, which in turn essentially enhanced the accumulation of anthocyanins, total flavonoids and soluble proanthocyanidins.

## Ectopic expression of *CsMYB5-1*/*CsMYB5-2* reduced anthocyanin/proanthocyanidin accumulation in flowers of the transgenic alfalfa

**Fig 5:**
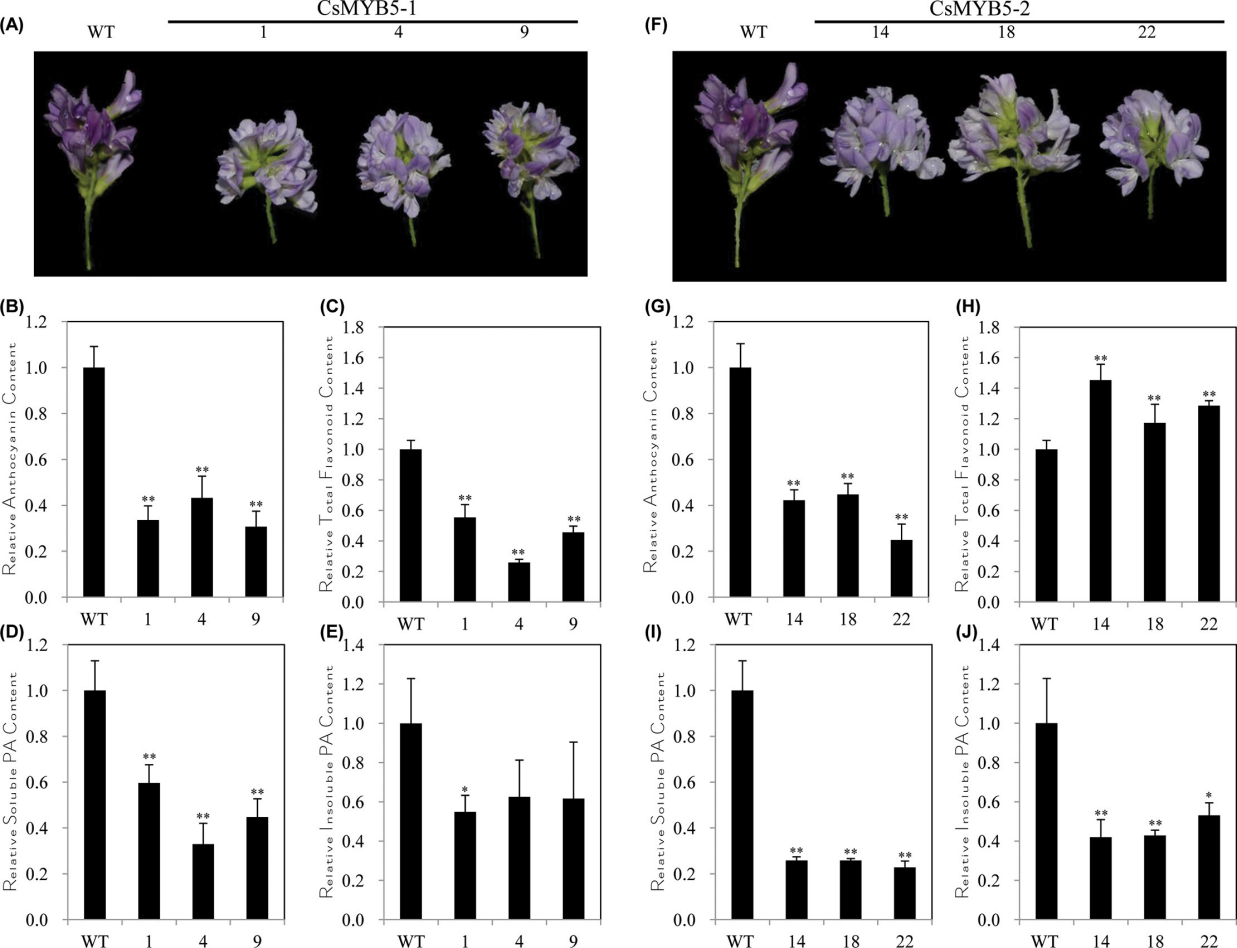
Morphology and flavonoid analyses in the flowers of *CsMYB5-1*/*CsMYB5-2* over-expressing alfalfa plants. (A, F) Flower phenotype of the transgenic alfalfa plants over-expressing *CsMYB5-1* (A), *CsMYB5-2* (F) in comparison with the wild type; (B, G) Relative anthocyanin content in flowers of the transgenic alfalfa plants over-expressing *CsMYB5-1* (B) or *CsMYB5-2* (G) in comparison with the wild type; (C, H) Relative total flavonoid content in flowers of the transgenic alfalfa plants over-expressing *CsMYB5-1* (C) or *CsMYB5-2* (H) in comparison with the wild type; (D, I) Relative soluble proanthocyanidin content in flowers of the transgenic alfalfa plants over-expressing *CsMYB5-1* (D) or *CsMYB5-2* (I) in comparison with the wild type; (E, J) Relative insoluble proanthocyanidin content in flowers of the transgenic alfalfa plants over-expressing *CsMYB5-1* (E) or *CsMYB5-2* (J) in comparison with the wild type. All the assays were performed with triplicates and the data are presented as mean ± SD, student’s *t*-test (n = 3, *P<0.05, **P<0.01).

The flower color of the transgenic alfalfa plants was much lighter than the wild type (Fig 5A and 5F), which was most likely due to the reduction of anthocyanin contents; we thus compared anthocyanin contents in flowers of both the transgenic and wild type alfalfa plants. It revealed that anthocyanin contents in flowers of the *CsMYB5-1* /*CsMYB5-2* transgenic alfalfa plants were only about 20% to 40% of the wild type (Fig 5B and 5G, S6 Table). Meanwhile, the total flavonoid contents were also reduced in the *CsMYB5-1* over-expressing lines, but increased in the *CsMYB5-2* over-expressing lines (Fig 5C and 5H, S7 Table).

The soluble proanthocyanidin contents in the transgenic lines reduced only 20% to 60% of the wild type (Fig 5D and 5I, S8 Table). Similar to the soluble proanthocyanidin portion, the insoluble proanthocyanidin contents reduced around 60% in the *CsMYB5-1* transgenic lines, and around 50% in all *CsMYB5-2* transgenic lines (Fig 5E and 5J, S8 Table). The above results illustrated that over-expression of *CsMYB5-1*/*CsMYB5-2* gene could reduce anthocyanin/proanthocyanidin accumulation in flowers. Therefore, the regulation of *CsMYB5-1*/*CsMYB5-2* gene in alfalfa appeared to be spatial-specific, which affected anthocyanins and proanthocyanidins differently in stems/leaves and flowers.

**Fig 6:**
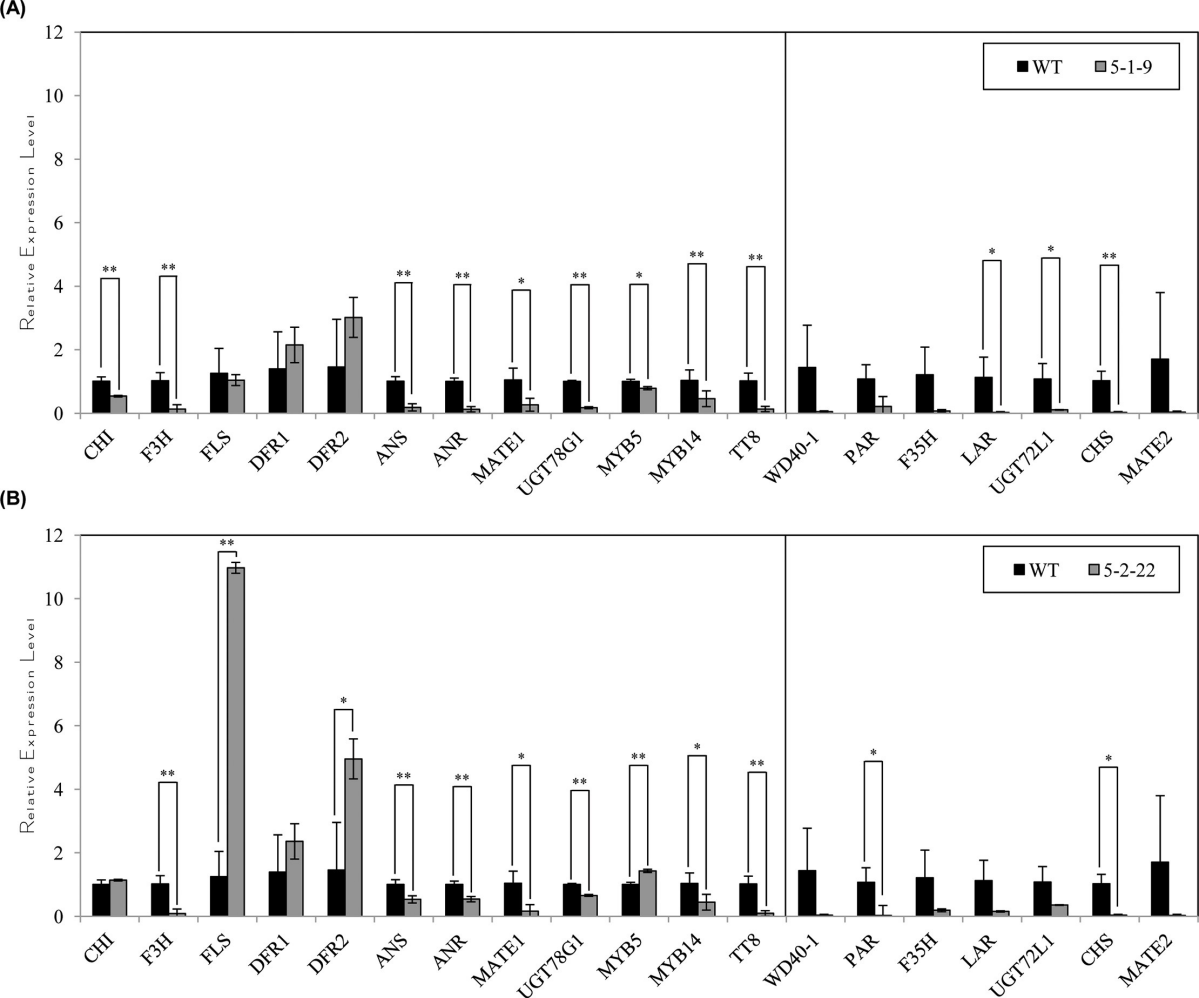
Relative expression level of flavonoid pathway genes in flowers of *CsMYB5-1*/*CsMYB5-2* over-expressing alfalfa lines. (A) Relative expression levels of flavonoid pathway genes in flowers of the alfalfa lines over-expressing *CsMYB5-1* gene; (B) Relative expression levels of flavonoid pathway genes in flowers of the alfalfa lines over-expressing *CsMYB5-2* gene. All the assays were performed with triplicates and the data are presented as mean ± SD, student’s *t*-test (n = 3, *P<0.05, **P<0.01).

qPCR analysis revealed that most of the pathway genes were reduced significantly in the *CsMYB5-1* and *CsMYB5-2* transgenic lines in comparison with the wild type in the flower (Fig 6, S9 Table), except *FLS*, *DFR1* and *DFR2* in line CsMYB5-1-9, and *CHI* and *DFR1* in line CsMYB5-2-22 (Fig 6, S9 Table). However, the expression levels of these genes were much lower than those in stems/leaves, in particular in line CsMYB5-1-9. By contrast, the expression level of *FLS* in line CsMYB5-2-22 was increased significantly by more than 7.8 fold, which was consistent with the highly increased total flavonoid content (Fig 5G).

In flowers, the expression levels of *CHS*, *LAR*, *UGT72L1*, *MATE2*, *PAR* and *WD40-1* were also detected and compared in the transgenic and wild type alfalfa lines. Note that the expression of these genes was not detected in stems/leaves. Among these genes, the expression levels of *CHS*, *LAR* and *UGT72L1* in line CsMYB5-1-9 (Fig 6A, S9 Table), and *CHS* and *PAR* in line CsMYB5-2-22 were significantly reduced by more than 90% than in the wild type (Fig 6B, S9 Table), indicating the down-regulation of these genes largely contributed to the decrease of anthocyanins/proanthocyanidins in flowers.

## Discussion

In the present study, we transformed two tea transcription factor genes *CsMYB5-1* and *CsMYB5-2* in forage crop alfalfa for flavonoid enhancement. Our results demonstrated that over-expression of *CsMYB5-1* and *CsMYB5-2* in alfalfa plants increased total flavonoids, anthocyanin and proanthocyanidin contents in stems/leaves (Fig 2). The increased anthocyanin contents resulted from the up-regulation of several enzyme genes (Figs 3 and 4), in particular *DFR2* and *ANS*, whereas the increase of proanthocyanidins is most likely due to the up-regulation of *ANR*, which is the key gene required for proanthocyanidin pathway [33].

Similarly, high *ANR* expression with increased proanthocyanidin content was also found in other plant species, including *T*. *repens* with *TaMYB14* over-expression [17], *M*. *truncatula* hairy roots with *MtPAR* and *AtTT2* over-expression [22, 34], grapevine hair roots with *VvMYBPA1* or *VvMYBPA2* over-expression [21] and poplar with *PtrMYB134* over-expression [35]. In addition, the expression levels of endogenous *MYB5*, *MYB14* and *TT8* were also up-regulated by *CsMYB5-1* in the transgenic alfalfa (Fig 3), which demonstrated that interaction between foreign and endogenous factors are necessary and specific, and successful stimulation of targeted flavonoids is dependent on such specific interaction [24].

In contrast to the increase of flavonoid compounds in stems/leaves, both anthocyanin and proanthocyanidin accumulation were reduced in the transgenic flowers over-expressing *CsMYB5-1*/*CsMYB5-2* (Fig 5). This could be explained by the suppressed expression of *ANS*, *CHS*, *MYB14*, *TT8* and *WD40-1* in the flowers (Fig 6), indicating that *CsMYB5-1*/*CsMYB5-2* could not interact with or induce these endogenous genes in this tissue. In another study, ectopic over-expression of grape *VvMYBPA1* in tobacco reduced anthocyanin level but accelerated proanthocyanidins in flowers [36]. And over-expression of another MYB transcript factor gene *AN2* in tobacco enhanced floral pigments but did not change proanthocyanidin level [37]. In these cases, the accumulation mechanism of anthocyanins and proanthocyanidins varied in different plant species, and it is likely that the difference in the regulation of MYB depend on plant species in floral tissues.

The MYB transcription factors in anthocyanin/proanthocyanidin pathway were divided into TT2 group and MYB5 group, respectively. TT2 group specifically regulate proanthocyanidins, whereas MYB5 group regulate both anthocyanins and proanthocyanidins. And this may explain the difference in regulation mechanism between CsMYB5-1 that is close to the TT2 group, and CsMYB5-2 that is closed to the MYB5 group [27]. Furthermore, *CsMYB5-1*/*CsMYB5-2* may activate anthocyanin/proanthocyanidin pathway genes in stems/leaves, which may repress expression of the same subsets of genes in flowers. The different phenotype between stems/leaves and flowers in transgenic lines suggest structure/tissue specificity of the flavonoid metabolic channel in different organs in alfalfa as found in other plant species [36].

Our study demonstrated that it is possible to improve the accumulation of anthocyanins and/or proanthocyanidins in stems/leaves of alfalfa by over-expression *CsMYB5-1* and *CsMYB5-2* genes. In the *CsMYB5-1*-over-expressing alfalfa, the expression level of *ANR* gene was up-regulated by more than 50 fold, which is still lower than that of *ANR* (increased by more than 400 fold) in the *AtTT2* over-expressing hairy roots of *M*. *truncatula*, in which massive proanthocyanidin accumulation was detected [26]. In several previous studies, foreign genes were transformed into alfalfa to promote proanthocyanidin accumulation, but failed to accumulate significant level of proanthocyanidins, which may be due to the un-sufficient expression level of endogenous *ANR* gene [22, 25, 26]. Therefore, the up-regulation of *ANR* could be the most important factor for the enhancement of proanthocyanidins in alfalfa. Furthermore, the bioysnthesis of proanthocyanidins requires additional processes associated with transport, sequestration and polymerization [38]. Even recent investigatio on *LAR* function had shed some light on proanthocyanidin polymerizaiton [39], but most of these steps still requre further investigation for the successful bioengineering of proanthocyanidin in alfalfa.

A number of flavonoid pathway genes were up-regulated by *CsMYB5-1*, but their expression levels were not as high as *DFR* for significant increase of anthocyanins. *CsMYB5-1* gene was expressed under the control of the 35S promoter, which was found to be an inefficient constitutive promoter in legumes such as alfalfa and *M*. *truncatula* [40]. Therefore, a stronger and more suitable promoter for transformation in alfalfa would be essential for the over-expression of target gene. In addition, *CsMYB5-1* could be utilized in other forage crop like clover or grass forage to promote proanthocyanidin accumulation in preventing pasture bloat.

## Conclusion

We over-expressed two tea MYB transcription factor genes *CsMYB5-1* and *CsMYB5-2* in alfalfa plants. The over-expression of these two genes activated the expression of flavonoid pathway genes and enhanced the accumulation of anthocyanins and proanthocyanidins in the foliage. These two genes could be applied in the genetic breeding of alfalfa with anti-bloat trait.

## Supporting information

S1 Fig Identification of transgenic alfalfa plants by PCR. Identification of transgenic alfalfa plants over-expressing *CsMYB5-1* (upper panel) and *CsMYB5-2* (lower panel).
(PDF)

S1 Table Gene-specific primers used in the present study. (PDF)

S2 Table Relative anthocyanin content in the stem/leaf of the transgenic alfalfa in comparison with the wild type. (PDF)

S3 Table Relative total flavonoid content in the stem/leaf of the transgenic alfalfa in comparison with the wild type. (PDF)

S4 Table Relative soluble and insoluble proanthocyanidin content in the stem/leaf of the transgenic alfalfa in comparison with the wild type. (PDF)

S5 Table Relative gene expression levels in the stem/leaf of the transgenic alfalfa in comparison with the wild type. (PDF)

S6 Table Relative anthocyanin content in the flower of the transgenic alfalfa in comparison with the wild type. (PDF)

S7 Table Relative total flavonoid content in the flower of the transgenic alfalfa in comparison with the wild type. (PDF)

S8 Table Relative soluble and insoluble proanthocyanidins content in the flower of the transgenic alfalfa in comparison with the wild type. (PDF)

S9 Table Relative gene expression levels in the flower of the transgenic alfalfa in comparison with the wild type. (PDF)
