# Supplementary material for: Ectopic expression of tea MYB genes alter spatial flavonoid accumulation in alfalfa (Medicago sativa)
Source: PLoS One. 2019 Jul 2;14(7):e0218336. doi: 10.1371/journal.pone.0218336 (PMC6605665; doi:10.1371/journal.pone.0218336)

S1 Fig. Identification of transgenic alfalfa plants by PCR. Identification of transgenic alfalfa plants over-expressing *CsMYB5-1* (upper panel) and *CsMYB5-2* (lower panel).

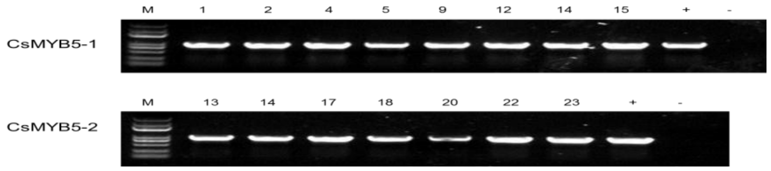

Supplement: S1 Fig — Identification of transgenic alfalfa plants over-expressing CsMYB5-1 (upper panel) and CsMYB5-2 (lower panel). (PDF) [file pone.0218336.s001.pdf]
