# Supplementary material for: Ectopic expression of tea MYB genes alter spatial flavonoid accumulation in alfalfa (Medicago sativa)
Source: PLoS One. 2019 Jul 2;14(7):e0218336. doi: 10.1371/journal.pone.0218336 (PMC6605665; doi:10.1371/journal.pone.0218336)
Supplement: S1 Table — (PDF) [file pone.0218336.s002.pdf]

S1 Table. Gene-specific primers used in the present study.

Primers names	Sequences (5'-3')
CsMYB5-1F	ATGGGGAGGAGTCCATGCTG
CsMYB5-1R	TCATGGCCAGTCCTCAGAATC
CsMYB5-2F	ATGGGAAGGGCTCCTTGTTGT
CsMYB5-2R	TCAGATCAACAAAGATTCAGC
MtCHIRTF	CACGCTGTTTCCCCTGATCT
MtCHIRTR	TCAACAACGCCGGTAATCTTG
MtF3HRTF	GTTCGAGTCGGGTTTCATTC
MtF3HRTR	GTCCATTCACCTCGTCGATT
MtDFR1RTF	TGTCCCTATGGAAGGCTGAC
MtDFR1RTR	TTCATTCTCAGGGTCCTTGG
MtDFR2RTF	TTCAAAAACCCTGGCAGAAC
MtDFR2RTR	GGTGGCATTGAAGGCATAAT
MtANSRTF	GGTTGGAAGGTGGAAGGTTA
MtANSRTR	CCCATTTGCCCTCATAGAAA
MtANRRTF	GCAGTTTCTATCGGGTTCAA
MtANRRTR	CTGAGGGTATCGTTTGCTGA
MtMATE1RTF	CAGAGAGCATAGCCGTGCAA
MtMATE1RTR	TTCTGCGCTTGGAGAAACCT
Actin2-rv	ACTCACACCGTCACCAGAATCC
Actin2-fw	TCAATGTGCCTGCCATGTATGT
MtFLS RTF	CACATGAGGTCGTGGTTGGA
MtFLS RTR	TTTGGCAGGGTTTTGATCATT
MtUGT78G1 RTF	GTTTTGGCATTCCCATTTGG
MtUGT78G1 RTR	GAGCCTCTGTAGCAATTTTTTTCAC
MtTT8 RTF	GCCAGCAACTAAGAGAACTCTATGAA
MtTT8 RTR	GGACGACGAGTTGGTGGATT
MtMYB5 RTF	ATCTTATCTGCTGCACCACC
MtMYB5 RTR	CCGTCTTGGTTTTGGGTGAA
MtMYB14 RTF	ACTCGGATTTTTCAACTGCATG
MtMYB14 RTR	ACTACATTCTGTCCAGTTCTTGAG
