# Supplementary material for: Ectopic expression of tea MYB genes alter spatial flavonoid accumulation in alfalfa (Medicago sativa)
Source: PLoS One. 2019 Jul 2;14(7):e0218336. doi: 10.1371/journal.pone.0218336 (PMC6605665; doi:10.1371/journal.pone.0218336)
Supplement: S2 Table — (PDF) [file pone.0218336.s003.pdf]

S2 Table. Relative anthocyanin content in the stem/leaf of the transgenic alfalfa in comparison with the wild type.

CsMYB5-1		
plant lines	average	SD
WT	1.00	0.2474
1	6.7143	1.0785
4	24	7.0812
9	16.8571	1.9325
CsMYB5-2		
plant lines	average	SD
WT	1.00	0.2474
14	12.2857	2.437
18	4.7143	0.4286
20	9.5714	0.9897
