# Supplementary material for: Ectopic expression of tea MYB genes alter spatial flavonoid accumulation in alfalfa (Medicago sativa)
Source: PLoS One. 2019 Jul 2;14(7):e0218336. doi: 10.1371/journal.pone.0218336 (PMC6605665; doi:10.1371/journal.pone.0218336)
Supplement: S3 Table — (PDF) [file pone.0218336.s004.pdf]

S3 Table. Relative total flavonoid contents in the stem/leaf of the transgenic alfalfa in comparison with the wild type.

CsMYB5-1		
plant lines	average	SD
WT	1.00	0.0307
1	1.7896	0.2091
4	1.1549	0.2415
9	1.8636	0.2844
CsMYB5-2		
plant lines	average	SD
WT	1.00	0.0307
14	1.6481	0.2273
18	1.6162	0.0862
20	1.77442	0.2781
