# Supplementary material for: Ectopic expression of tea MYB genes alter spatial flavonoid accumulation in alfalfa (Medicago sativa)
Source: PLoS One. 2019 Jul 2;14(7):e0218336. doi: 10.1371/journal.pone.0218336 (PMC6605665; doi:10.1371/journal.pone.0218336)
Supplement: S4 Table — (PDF) [file pone.0218336.s005.pdf]

S4 Table. Relative soluble and insoluble proanthocyanidin contents in the flower of the transgenic alfalfa in comparison with the wild type.

CsMYB5-1			CsMYB5-2		
Soluble proanthocyanidins			Soluble proanthocyanidins		
plant lines	average	SD	plant lines	average	SD
WT	1.00	0.0211	WT	1.00	0.0211
1	1.3715	0.0717	14	1.4676	0.0805
4	1.5417	0.1469	18	1.2431	0.0535
9	1.6192	0.0852	20	1.2616	0.014
Insoluble proanthocyanidins			Insoluble proanthocyanidins		
plant lines	average	SD	plant lines	average	SD
WT	1.00	0.2677	WT	1.00	0.2677
1	0.9135	0.1566	14	1.1289	0.0985
4	0.8802	0.1396	18	1.3291	0.1637
9	0.9837	0.2578	20	1.5076	0.4377
