# Supplementary material for: Ectopic expression of tea MYB genes alter spatial flavonoid accumulation in alfalfa (Medicago sativa)
Source: PLoS One. 2019 Jul 2;14(7):e0218336. doi: 10.1371/journal.pone.0218336 (PMC6605665; doi:10.1371/journal.pone.0218336)
Supplement: S5 Table — (PDF) [file pone.0218336.s006.pdf]

S5 Table. Relative gene expression levels in the stem/leaf of the transgenic alfalfa in comparison with the wild type.

	WT		5-1-9	
Gene name	average	SD	average	SD
CHI	1.0019	0.0749	0.7003	0.04
F3H	1.1404	0.6336	15.1797	4.6443
FLS	1.727	1.5038	21.7505	7.2744
DFR1	1.0057	0.1272	4.3251	0.7018
DFR2	1.4609	1.4784	10230.25	1405.459
ANS	1.00	0.00	666.5122	174.5139
ANR	1.0352	0.3785	57.3433	6.0811
MATE1	1.0012	0.0599	4.3033	2.577
UGT78G1	1.0123	0.1994	41.9932	8.1189
MYB5	1.01	0.19	16.59	3.09
MYB14	1.01	0.14	99.08	8.67
TT8	1.53	1.69	72.48	21.73
	WT		5-2-22	
Gene name	average	SD	average	SD
CHI	1.0028	0.0903	3.3241	0.2246
F3H	1.0713	0.5124	1.3467	0.5438
FLS	1.1196	0.6311	1.2527	0.6619
DFR1	1.0022	0.0816	1.1079	0.1075
DFR2	1.007	0.1482	5.3451	0.939
ANS	1.005	0.1251	6.0498	1.6261
ANR	1.0511	0.4262	2.6888	0.1737
MATE1	1.0061	0.1325	0.1866	0.0731
UGT78G1	1.0042	0.1149	0.5008	0.0395
MYB5	1.0112	0.1875	1.36	0.2247
MYB14	1.0068	0.1412	1.775	0.4528
TT8	1.0068	0.1413	2.8683	0.6623
