# Supplementary material for: Ectopic expression of tea MYB genes alter spatial flavonoid accumulation in alfalfa (Medicago sativa)
Source: PLoS One. 2019 Jul 2;14(7):e0218336. doi: 10.1371/journal.pone.0218336 (PMC6605665; doi:10.1371/journal.pone.0218336)
Supplement: S6 Table — (PDF) [file pone.0218336.s007.pdf]

S6 Table. Relative anthocyanin contents in the flower of the transgenic alfalfa in comparison with the wild type.

CsMYB5-1		
plant lines	average	SD
WT	1	0.0912
1	0.3361	0.0618
4	0.4330	0.0941
9	0.3075	0.0672
CsMYB5-2		
plant lines	average	SD
WT	1	0.1038
14	0.4224	0.0450
18	0.4479	0.0468
22	0.2495	0.0691
