# Supplementary material for: Ectopic expression of tea MYB genes alter spatial flavonoid accumulation in alfalfa (Medicago sativa)
Source: PLoS One. 2019 Jul 2;14(7):e0218336. doi: 10.1371/journal.pone.0218336 (PMC6605665; doi:10.1371/journal.pone.0218336)
Supplement: S7 Table — (PDF) [file pone.0218336.s008.pdf]

S7 Table. Relative total flavonoid contents in the flower of the transgenic alfalfa in comparison with the wild type.

CsMYB5-1		
plant lines	average	SD
WT	1.00	0.0584
1	0.5541	0.0841
4	0.2590	0.0202
9	0.4562	0.0403
CsMYB5-2		
plant lines	average	SD
WT	1.00	0.0584
14	1.4536	0.1033
18	1.1732	0.1215
20	1.2850	0.0330
