# Supplementary material for: Ectopic expression of tea MYB genes alter spatial flavonoid accumulation in alfalfa (Medicago sativa)
Source: PLoS One. 2019 Jul 2;14(7):e0218336. doi: 10.1371/journal.pone.0218336 (PMC6605665; doi:10.1371/journal.pone.0218336)
Supplement: S8 Table — (PDF) [file pone.0218336.s009.pdf]

**S8 Table. Relative soluble and insoluble proanthocyanidin contents in the flower of the transgenic alfalfa in comparison with the wild type.**

| CsMYB5-1 | | | CsMYB5-2 | | |
|---|---|---|---|---|---|
| Soluble proanthocyanidins | | | Soluble proanthocyanidins | | |
| plant lines | average | SD | plant lines | average | SD |
| WT | 1.00 | 0.1296 | WT | 1.00 | 0.1296 |
| 1 | 0.5965 | 0.0793 | 14 | 0.2588 | 0.0152 |
| 4 | 0.3289 | 0.0912 | 18 | 0.2588 | 0.0076 |
| 9 | 0.4474 | 0.08004 | 22 | 0.22807 | 0.0273 |
| | | | | | |
| Insoluble proanthocyanidins | | | Insoluble proanthocyanidins | | |
| plant lines | average | SD | plant lines | average | SD |
| WT | 1.00 | 0.2278 | WT | 1.00 | 0.2278 |
| 1 | 0.5491 | 0.0836 | 14 | 0.4196 | 0.0892 |
| 4 | 0.625 | 0.1877 | 18 | 0.4286 | 0.0268 |
| 9 | 0.6161 | 0.2882 | 22 | 0.5313 | 0.0633 |
