# Supplementary material for: Ectopic expression of tea MYB genes alter spatial flavonoid accumulation in alfalfa (Medicago sativa)
Source: PLoS One. 2019 Jul 2;14(7):e0218336. doi: 10.1371/journal.pone.0218336 (PMC6605665; doi:10.1371/journal.pone.0218336)
Supplement: S9 Table — (PDF) [file pone.0218336.s010.pdf]

S9 Table. Relative gene expression levels in the flower of the transgenic alfalfa in comparison with the wild type.

	WT		5-1-9	
Gene name	average	SD	average	SD
CHI	1.0061	0.1390	0.5378	0.0271
F3H	1.0224	0.2557	0.1285	0.1419
FLS	1.2532	0.7897	1.0428	0.1708
DFR1	1.3947	1.1684	2.1493	0.5585
DFR2	1.4576	1.4998	3.0160	0.6294
ANS	1.0066	0.1448	0.1845	0.1123
ANR	1.0035	0.1049	0.1242	0.0841
MATE1	1.0436	0.3770	0.2674	0.2021
UGT78G1	1.0003	0.0320	0.1739	0.0313
MYB5	1.0016	0.0695	0.7889	0.0500
MYB14	1.0333	0.3318	0.4559	0.2483
TT8	1.0194	0.2440	0.1334	0.0848
WD40-1	1.4392	1.3330	0.0475	0.0268
PAR	1.0735	0.4539	0.2117	0.3123
F35H	1.2132	0.8694	0.0709	0.0454
LAR	1.1255	0.6401	0.0281	0.0199
UGT72L1	1.0764	0.4897	0.1068	0.0098
CHS	1.0272	0.2944	0.0272	0.0239
MATE2	1.6693	1.9924	0.00	0.0000
	WT		5-2-22	
Gene name	average	SD	average	SD
CHI	1.0061	0.1390	1.1387	0.0225
F3H	1.0224	0.2557	0.0884	0.0251
FLS	1.2532	0.7897	10.9725	0.3938
DFR1	1.3947	1.1684	2.3580	0.6995
DFR2	1.4576	1.4998	4.9555	0.8375
ANS	1.0066	0.1448	0.5326	0.0533
ANR	1.0035	0.1049	0.5387	0.0776
MATE1	1.0436	0.3770	0.1638	0.0784
UGT78G1	1.0003	0.0320	0.6575	0.0705
MYB5	1.0016	0.0695	1.4310	0.0251
MYB14	1.0333	0.3318	0.4442	0.1411
TT8	1.0194	0.2440	0.0918	0.0761
WD40-1	1.4392	1.3330	0.0349	0.0043
PAR	1.0735	0.4539	0.0303	0.0247
F35H	1.2132	0.8694	0.1883	0.2396
LAR	1.1255	0.6401	0.1533	0.0367

UGT72L1	1.0764	0.4897	0.3535	0.1259
CHS	1.0272	0.2944	0.0392	0.0308
MATE2	1.6693	1.9924	0.00	0.00
